# Tomato Leaf Disease Identification Framework FCMNet Based on Multimodal Fusion

**DOI:** 10.3390/plants14152329

**Published:** 2025-07-27

**Authors:** Siming Deng, Jiale Zhu, Yang Hu, Mingfang He, Yonglin Xia

**Affiliations:** 1 College of Bangor, Central South University of Forestry and Technology, Changsha 410004, China; 20227715@csuft.edu.cn; 2 College of Electronic Information & Physics, Central South University of Forestry and Technology, Changsha 410004, China; 20231100278@csuft.edu.cn (J.Z.); 20231100398@csuft.edu.cn (Y.H.); t20162306@csuft.edu.cn (M.H.); 3 College of Computer & Mathematics, Central South University of Forestry and Technology, Changsha 410004, China

**Keywords:** tomato leaf disease identification, multimodal fusion, FCMNet, FGAM, CVLA, MSCOA

## Abstract

Precisely recognizing diseases in tomato leaves plays a crucial role in enhancing the health, productivity, and quality of tomato crops. However, disease identification methods that rely on single-mode information often face the problems of insufficient accuracy and weak generalization ability. Therefore, this paper proposes a tomato leaf disease recognition framework FCMNet based on multimodal fusion, which combines tomato leaf disease image and text description to enhance the ability to capture disease characteristics. In this paper, the Fourier-guided Attention Mechanism (FGAM) is designed, which systematically embeds the Fourier frequency-domain information into the spatial-channel attention structure for the first time, enhances the stability and noise resistance of feature expression through spectral transform, and realizes more accurate lesion location by means of multi-scale fusion of local and global features. In order to realize the deep semantic interaction between image and text modality, a Cross Vision–Language Alignment module (CVLA) is further proposed. This module generates visual representations compatible with Bert embeddings by utilizing block segmentation and feature mapping techniques. Additionally, it incorporates a probability-based weighting mechanism to achieve enhanced multimodal fusion, significantly strengthening the model’s comprehension of semantic relationships across different modalities. Furthermore, to enhance both training efficiency and parameter optimization capabilities of the model, we introduce a Multi-strategy Improved Coati Optimization Algorithm (MSCOA). This algorithm integrates Good Point Set initialization with a Golden Sine search strategy, thereby boosting global exploration, accelerating convergence, and effectively preventing entrapment in local optima. Consequently, it exhibits robust adaptability and stable performance within high-dimensional search spaces. The experimental results show that the FCMNet model has increased the accuracy and precision by 2.61% and 2.85%, respectively, compared with the baseline model on the self-built dataset of tomato leaf diseases, and the recall and F1 score have increased by 3.03% and 3.06%, respectively, which is significantly superior to the existing methods. This research provides a new solution for the identification of tomato leaf diseases and has broad potential for agricultural applications.

## 1. Introduction

As a vegetable crop extensively cultivated worldwide, tomato not only occupies a core position in the agricultural production system of many countries, but also is an important raw material for the food processing industry [1]. It is vulnerable to a variety of diseases in the growth process, especially in the large-scale intensive planting mode, and the occurrence and spread of diseases often lead to a large area of yield reduction, which seriously affects the yield of crops, and then causes significant economic losses to the agricultural production system [2]. According to statistics, the global annual yield loss caused by tomato diseases is as high as millions of tons, posing a significant challenge to the sustainable advancement of agriculture [3]. Therefore, the realization of efficient and accurate identification of tomato diseases has important practical significance for ensuring crop yield, improving product quality and maintaining agricultural ecological security [4].

At present, the traditional tomato leaf disease identification mainly relies on manual visual identification and empirical judgment, which has significant limitations such as strong subjectivity, low efficiency, and limited recognition accuracy [5]. With the rapid development of computer vision and deep learning technology, plant disease recognition method based on deep neural networks is gradually being widely used in the field of agriculture, showing stronger feature extraction ability and higher recognition accuracy [6]. The introduction of the deep learning method into tomato leaf disease classification research not only helps to improve the automation and intelligence level of disease detection, but also provides effective technical support for promoting the development of precision agriculture and smart agriculture [7]. Yang et al. put forward a systematic review study on the application of deep learning technology in the cotton industry. The study pointed out that the traditional spectral imaging and machine learning methods had problems of poor adaptability and limited accuracy in complex agricultural environments, while the deep learning method showed significant advantages in improving the level of agricultural automation and intelligence by virtue of its excellent feature extraction ability and adaptive analysis ability [8]. Dinesh et al. introduced a deep learning approach aimed at detecting diseases in citrus leaves. Their method integrated DeepOverlay L-UNet with VGG-RefineNet to effectively perform segmentation of lesion regions, disease classification and severity visualization through a three-stage process, effectively improving the detection accuracy and interpretability [9]. Zhang et al. proposed a small sample detection model of tea disease MAF-MixNet based on the fusion of mixed attention and multi-path features. By introducing the Ma branch of context feature extraction (MA-Branch) and the multi-path fusion module of local global information enhancement (MAFM), the model can achieve high-precision detection under the condition of limited samples and effectively alleviate the bottleneck problem of feature extraction caused by data scarcity [10]. Sagar et al. presented an improved ResNet50-based approach for detecting grape downy mildew and powdery mildew in both leaves and fruits. They integrated batch normalization into the network structure, significantly boosting the model’s stability and detection performance; classification accuracy of 95% was achieved on the image dataset of five types of grape diseases, and the F1 score was 95%, which was significantly better than the traditional method [11]. Rajeena et al. introduced an approach utilizing the EfficientNet model to identify diseases in maize leaves. The overall process includes image acquisition, preprocessing and classification. In the preprocessing stage, the method uses image reading, scaling, and data enhancement to improve the generalization ability of the model, and uses the idea of transfer learning to fine-tune the parameters of EfficientNet structure. At the same time, DenseNet and ResNet models are introduced for comparative analysis to evaluate the performance of different networks in disease feature extraction, so as to build a more efficient automatic disease detection system [12]. Despite substantial advancements achieved by these approaches in recognizing plant diseases, most of them still rely on single-mode data input, such as using only visible image or static image features, which may limit the performance of the model in the face of complex environmental conditions (such as light changes, leaf occlusion, weak symptoms in the early stage of disease, etc.). In addition, the generalization ability and robustness of existing methods are still insufficient when dealing with new diseases or data samples are scarce.

In recent years, multimodal technology has gradually attracted the attention of researchers, especially in the combination of images, texts and other information sources. Through the fusion of multimodal data, disease feature information can be more comprehensively captured [13]. Wang et al. proposed a multimodal maize disease classification model, WCG-VMamba, to improve the recognition performance through image and text feature alignment and fusion. This model combines the image coding network WAVM with the Cross-modal Attention Mechanism (CMAT), and introduces the Gaussian random walk duck swarm algorithm (GRW-DSA) to adjust the learning rate, achieving a recognition accuracy better than the mainstream model on multiple datasets [14]. Li et al. proposed a multimodal tomato leaf disease recognition model PDC-VLD based on open vocabulary target detection, combined with visual transformer and convolution pyramid module to extract disease features, and improved the adaptability in low-sample scenarios through context guidance mechanism. The model can identify new diseases without manual labeling, and is superior to the existing open identification model in many indicators, showing good generalization ability and application prospects [15]. Hu et al. proposed a tomato disease segmentation framework, LVF, which fuses image and text information, and uses the combined text and text presegmentation strategy. This method combines the probability difference fusion network to suppress high-frequency noise, extracts effective image information by enhancing the feature extraction and threshold filtering module, and introduces the multi-scale cross-nested network to fuse text semantics, so as to improve the segmentation accuracy of the model for the lesion area under complex background [16].

Although these studies offer significant insights into agricultural tomato disease detection, three primary challenges remain in the domain of multimodal tomato disease recognition research:

(a) The complexity of image in field environment seriously affects the effective modeling of lesion area. Multimodal tomato disease recognition usually relies on image modality as one of the important inputs, but in field scenes such as natural light, leaf overlap, occlusion and background clutter, it is often difficult to distinguish the diseased area from the healthy area, which makes it difficult for the model to focus on the key diseased area, thus affecting the recognition accuracy.
(b) There is inconsistency between the expression form and semantic structure between image and text modalities, which limits the effective fusion of cross-modal features. In the task of tomato disease recognition, text modality (such as disease description or expert annotation sentence) contains rich semantic information, but the differences in structure, distribution, and representation between the text modality and image modality make it difficult to achieve high-quality alignment at the feature level, which reduces the recognition ability of the multimodal model.
(c) The multimodal model has a complex structure and many parameters, and the training process is strongly dependent on resources, and the adjustment of learning rate has a significant impact on the performance of the model. In multimodal tomato disease recognition, the model needs to process image and text information at the same time, and the training difficulty is significantly higher than that of the single-mode model, which is prone to slow convergence speed, large accuracy fluctuation and unstable training process, which restricts its application in large-scale agricultural scenes.

To address the issue of complicated backgrounds affecting image feature extraction, Liu et al. developed a rice disease identification approach named PSOC-DRCNet. This method utilizes a dual-mode attention mechanism (DMA) to emphasize crucial lesion regions, along with a residual adaptive block (RAB) designed to enhance edge feature extraction capability. Consequently, their approach effectively reduces problems arising from background noise and unclear boundaries, significantly improving the robustness and practical performance of the model under complex environmental conditions [17]. Fang et al. introduced HCA-MFFNet, a novel network architecture for recognizing corn diseases amidst complicated backgrounds. Their method employs the hard coordinate attention (HCA) module to strengthen lesion-related feature representation, integrating multi-channel feature fusion and depthwise separable convolutions. These enhancements effectively mitigate background disturbances, substantially boosting model accuracy in realistic agricultural scenarios [18]. Although these two methods improve accuracy under noisy conditions, they have a large depth and many parameters, which limit lightweight deployment. However, our work addresses the issues of efficiency and robustness by incorporating frequency-domain guidance.

Aiming at the fusion problem caused by the semantic and structural differences between the image and text modality, Xu et al. developed a tomato leaf disease retrieval method named LAFANet, which leverages both image and text modalities. This method uses vit and Bert to extract the deep semantic features of image and text, respectively, and enhances the semantic interaction between text and image by the learning fusion attention mechanism (LFA), which effectively improves the quality of cross-modal feature alignment. Meanwhile, the adversarial negative sample screening mechanism (FNE-ANS) is introduced to further optimize the triplet training strategy, thereby alleviating the interference caused by semantic inconsistency between modalities. This research provides a new idea for the task of cross-modal disease identification to efficiently integrate the image and text information, and enhances the model’s capability in comprehending and accurately aligning semantic information related to diseases [19]. Yang et al. proposed a triple contrast learning (TCL) method to solve the problem of semantic inconsistency between text and text modes. On the basis of traditional cross-modal contrast learning (CMA), the intra-modal contrast target was added to enhance the synmodal semantic aggregation. At the same time, TCL enhances the effect of structural feature alignment by maximizing the mutual information between local and global representations of text and text, so as to strengthen the cross-modal feature fusion [20]. Although the above methods perform well in improving the image text fusion effect, there are still problems such as strong dependence on high-quality image text pairing, and insufficient robustness under semantic inconsistency or noisy data, which affect the generalization ability of the model in the actual scene. Our Cross Vision–Language Alignment (CVLA) module builds on these ideas, introducing probability-weighted fusion to enhance resilience against noisy or imperfect pairings.

In order to find the optimal learning rate and maintain the stability of the training process, Dai et al. introduced an innovative optimization approach called the annealing integrated sparrow optimization algorithm (AISOA). This algorithm integrates the random search capabilities of the sparrow optimization technique with the controlled, progressive constraints of an annealing process. Consequently, AISOA effectively prevents models from becoming trapped in local optima and significantly improves the detection accuracy of indistinct boundary features [21]. Zhao et al. proposed a language and vision fusion method LVR for rice field disease segmentation in complex environments. In this method, adaptive gradient enhancement algorithm (SAGE) is introduced to dynamically adjust the learning rate according to the training process, which can effectively accelerate the convergence speed of the model and improve the training efficiency. In addition, the combination of CNN and transformer architecture improves the performance stability of the model in complex environments and provides an efficient optimization strategy for multimodal disease segmentation in agricultural scenes [22]. Although AISOA and SAGE show some advantages in optimizing the learning rate and accelerating the convergence of the model, they are sensitive to the initial parameters and may need to be readjusted when generalized to different tasks, with limited adaptability. Multi-strategy Improved Coati Optimization Algorithm (MSCOA) overcomes these limitations by combining Good Point Set initialization and Golden Sine-based global search, ensuring faster convergence and better adaptability.

The main contributions of this paper are summarized as follows:

(1) To effectively train our proposed network, we built a dataset comprising 6994 images of tomato leaf diseases, each annotated with corresponding textual descriptions to facilitate enhanced model learning.
(2) A Fourier-guided Attention Mechanism (FGAM) is proposed. This mechanism introduces spatial and channel attention into the Fourier frequency domain, and enhances the stability and anti-noise ability of feature expression through Fourier transform, thereby improving the perception effect of lesion areas in complex backgrounds. The FGAM module divides the input features locally and globally through the separation factor, processes them using convolution and spectrum information, respectively, and fuses semi-global features to realize the information complementarity between multi-scale features. As far as we know, this is the first time that the Fourier domain guidance mechanism is systematically embedded into the space channel attention structure and applied to the task of plant disease recognition, which effectively enhances the robustness and detail recognition ability of the model.
(3) A Cross Vision–Language Alignment module (CVLA) was proposed to align image and text features more effectively, improve the information fusion ability between different modes, and enhance the accuracy of tomato disease identification. In this module, the high-resolution image is mapped into a representation that can be embedded and aligned with Bert through block partition and visual feature coding, and the probability-weighting mechanism is used to realize the deep fusion of visual and linguistic information, which enhances the model’s ability to understand the cross-modal semantic association.
(4) A Multi-strategy Improved Coati Optimization Algorithm (MSCOA) is proposed. MSCOA integrates the Good Point Set method and the Golden Sine Algorithm into the Coati Optimization Algorithm (COA). The introduction of Good Point Set improves the uniformity of population initialization and ensures the full coverage of search space. Combined with the Golden Sine Algorithm, the global search ability is enhanced, its full coverage characteristics and golden section strategy are used to effectively avoid falling into local optimization, and the convergence speed is accelerated. At the same time, MSCOA shows excellent adaptability in high-dimensional space optimization.
(5) The experimental results obtained from a self-constructed dataset of tomato leaf diseases revealed that FCMNet outperformed the baseline model ResNet50, enhancing accuracy and precision by 2.61% and 2.85%, respectively. Additionally, recall and F1 score were increased by 3.03% and 3.06%, respectively, reflecting an overall improvement in performance, which provided a method support for the rapid identification and detection of tomato leaf disease, and the overall performance was better than the baseline model. The multimodal recognition model can effectively integrate image and text information, improve the accuracy and stability of tomato disease recognition, and provide more reliable technical support for intelligent diagnosis of agricultural diseases.

## 2. Materials and Methods

### 2.1. Multimodal Dataset for Tomato Leaf Disease Retrieval

#### 2.1.1. Initial Dataset Selection and Preprocessing

During the creation of the multimodal dataset for tomato leaf disease retrieval, data was gathered from the tomato cultivation area at the Plant Protection Institute, Hunan Academy of Agricultural Sciences. This collection encompassed various disease categories under diverse environmental scenarios. The collected image data includes tomato leaves under different weather conditions. The acquisition device is Sony A7M4(Tokyo, Japan.), and the camera has 33 million pixels. Different lighting conditions (such as strong light, cloudy and rainy days) and time periods (such as morning, noon and dusk) are taken into account to ensure the diversity and integrity of disease characteristics. A total of 6,994 tomato leaf images were collected, covering healthy leaves and six typical diseases (Bacterial Leaf Spot, Early Blight, Late Blight, Leaf Mold, Leaf Blight and Yellow Leaf Curl). In order to ensure that the model can effectively process and train these image data, we preprocessed the dataset. Due to the different image sizes, in order to unify the input data format, we adjusted the resolution of all images to 224 × 224 pixels to ensure that each image is consistent in spatial dimension, which is helpful for subsequent feature extraction and model training. To maintain scientific rigor and reliability during model training and assessment, the image dataset is split into three subsets—training, validation, and testing—following an 8:1:1 ratio.

#### 2.1.2. Textual Data Generation and Annotation

**Figure 1:**
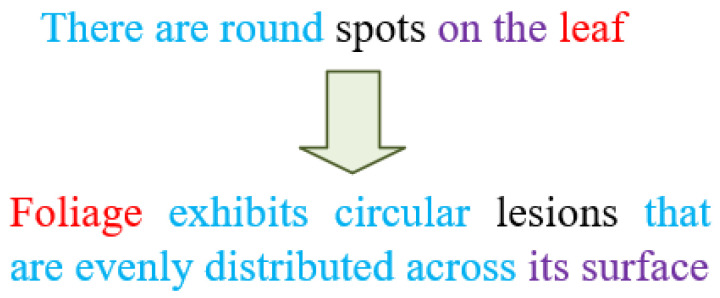
Text expansion and replacement process.

**Figure 2:**
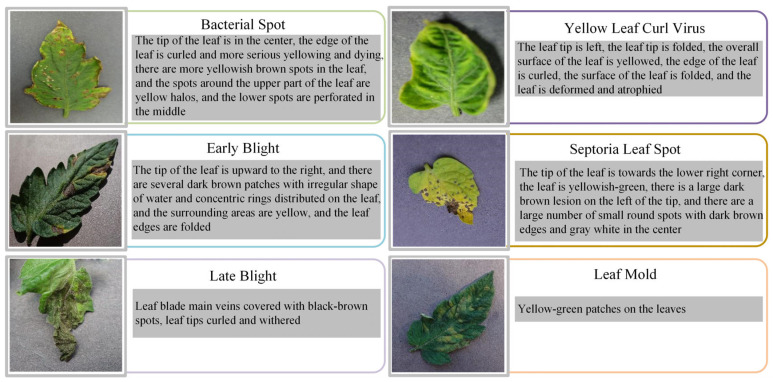
Partial data samples.

During the development of the multimodal dataset comprising tomato leaf disease images and corresponding textual annotations, we collaborated with experts from the Hunan Academy of Agricultural Sciences for detailed labeling. To construct the in-house dataset, each image was independently labeled by two plant pathology experts according to a standardized taxonomy of six disease classes and a healthy class. In approximately five percent of cases, the experts disagreed and those discrepancies were resolved in a consensus meeting to ensure that ambiguous symptoms—such as overlapping lesions of Early and Late Blight—were classified according to predefined criteria including lesion shape, margin necrosis, and sporangia presence. Inter-annotator agreement measured by Cohen’s Kappa exceeded 0.87 across all categories, demonstrating high labeling consistency. To mitigate bias and further reduce manual inspection errors, we introduced an initial machine-assisted screening step in which a pretrained convolutional neural network flagged images with a softmax confidence score below 0.6 for expert re-review, thereby focusing human effort on the most challenging samples. This rigorous multi-stage classification protocol leverages human expertise while minimizing subjective variability and provides a reliable foundation for automated disease identification. These experts systematically reviewed images representing each of the six tomato leaf diseases, providing thorough descriptions that highlighted visual characteristics such as lesion patterns, color variations, lesion and leaf locations, lesion quantity, and other semantic nuances. The comprehensive annotations were designed specifically to establish precise correspondences between image and textual data. Our annotation protocol strictly adhered to uniform guidelines across the six disease categories, emphasizing the detailed and consistent representation of leaf characteristics, including patterns, colors, lesion positions, leaf positions, quantities, and additional descriptive features, in order to ensure the normalization and richness of the description text and avoid the overconcentration of specific words. Through methods such as synonym replacement and sentence expansion, we generated multiple varied yet semantically similar textual descriptions. These alternative texts were employed to annotate images sharing similar visual features within the same disease category, for example, replacing “spots” with “lesions” and “leaves” with “foliage”. For the sentence “there are round spots on the leaves”, we can expand it to “Foliage exhibits circular lesions that are evenly distributed across its surface”. This process is shown in Figure 1. Using the approaches described above, we successfully developed a high-quality multimodal dataset consisting of paired images and textual annotations for tomato leaf diseases, illustrated in Figure 2.

### 2.2. Tomato Disease Identification Framework

**Figure 3:**
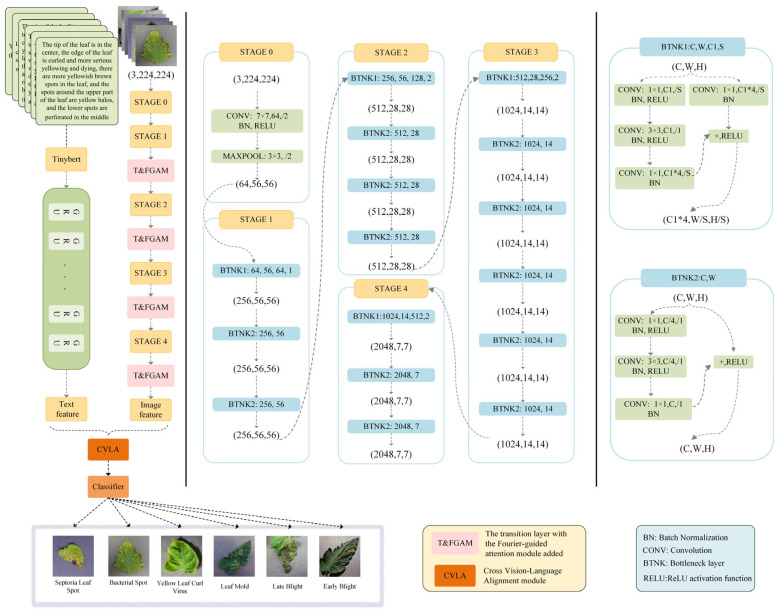
FCMNet framework.

In this paper, a multimodal fusion recognition framework FCMNet based on image and text is proposed to solve the problem of recognition and classification of tomato leaf diseases in complex agricultural environment. The proposed framework is primarily built upon the ResNet50 architecture, supplemented by three key modules specifically designed to boost the model’s performance. Figure 3 illustrates the overall structure of the framework. Among these modules, the Fourier-guided Attention Mechanism (FGAM) is incorporated to enhance lesion localization and strengthen feature extraction capabilities. The Cross Vision–Language Alignment module (CVLA) is used to promote the feature fusion of image and text features; The Multi-strategy Enhanced Coati Optimization Algorithm (MSCOA) is employed to optimize the learning rate, thereby accelerating convergence during training and enhancing overall training efficiency.

#### 2.2.1. Fourier-Guided Attention Mechanism (FGAM)

By imitating the focusing ability of human vision, the attention mechanism notably enhances deep learning models’ capabilities, particularly in tasks related to computer vision and natural language processing. Its function is to automatically assign different weights to different parts of the input data, so as to help the model focus on key features and ignore irrelevant parts [23]. Spatial attention [24] and channel attention [25] are typical and widely used attention mechanisms. By applying weighted adjustments to feature maps separately along spatial and channel dimensions, the model’s overall performance is effectively enhanced.

Although spatial and channel attention mechanisms have achieved good results in many tasks, they also have some disadvantages, especially in complex backgrounds or high-noise environments. Attention mechanisms typically rely on weighted calculations involving local and global features, which may cause inaccuracies in attention distribution under significant noise interference, hindering the precise capture of detailed features and reducing model accuracy and robustness. To address this limitation, we introduce the Fourier-guided Attention Mechanism (FGAM), which integrates spatial and channel attention strategies within the Fourier transform framework. The Fourier transform is capable of converting images from the spatial domain into the frequency domain, effectively suppressing noise and extracting more stable frequency-domain features, so as to provide more accurate input for the attention mechanism and improve performance in complex backgrounds [26].

**Figure 4:**
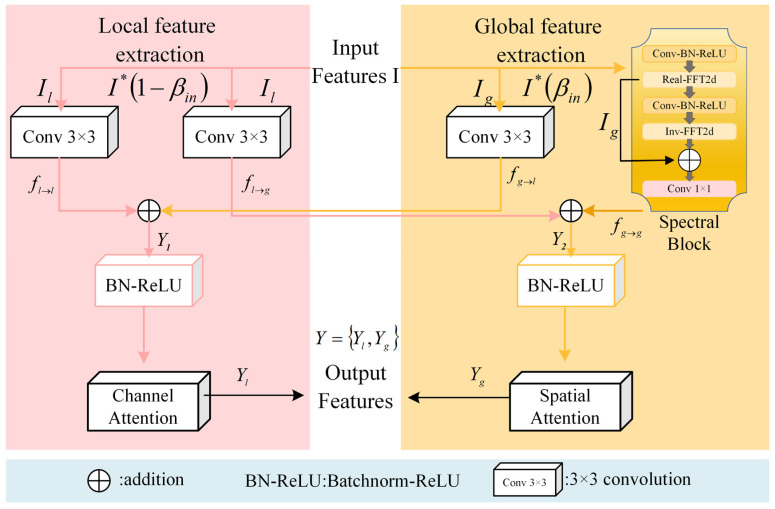
Fourier-guided Attention Mechanism. Note: Conv-BN-ReLu stands for the combination of convolution, batch normalization, and ReLU activation; Real-FFT2d is responsible for performing a 2D Fast Fourier Transform of real features; Inv-FFT2d implements the inverse transform from the Fourier domain to the real domain; and Conv 1 × 1 is used for 1 × 1 convolution to help adjust the dimensionality of the feature mappings or to fuse channel information.

As shown in Figure 4, our model introduces a Fourier guidance module for extracting global and local features. Given an input feature map $I\in R^{H\times W\times C}$, where H×W denotes spatial resolution and C denotes channel count, a separation factor determines the proportion of the feature map utilized for global feature extraction $I_{g}$, while the remaining portion is dedicated to local feature extraction $I_{l}$. The separation factor, represented as $\beta_{\mathrm{in}}$, falls within the interval $\left[0,\;1\right]$.

(1) $$I_{g}\in R^{H\times W\times \left(\beta_{in}\right)C}$$

(2) $$I_{l}\in R^{H\times W\times \left(1-\beta_{in}\right)C}$$

(3) $$\beta_{in}=\frac{S_{global}}{S_{total}}$$

where $S_{global}$ is the spatial region used for global feature extraction, and $S_{total}$ is the total area of the feature map.

To effectively capture global information, the input feature $I_{g}$ is processed through a spectral block, which employs the efficient Cooley–Tukey algorithm [27] for Fast Fourier Transform. This spectral block transforms spatial-domain features into the frequency (spectral) domain, allowing global updating of the spectral features. Subsequently, the updated frequency-domain data is converted back into the spatial domain. Among them, the two-dimensional Fast Fourier Transform maps the input characteristic image $i\left[m,n\right]$ to the frequency domain to represent $I_{g}\left[u,v\right]$, and its computational procedure is expressed as follows:

(4) $$I_{g}[u,v]=\sum_{n=0}^{M-1}\sum_{m=0}^{N-1}i[m,n]\cdot e^{-j2\pi \left(\frac{un}{M}+\frac{vm}{N}\right)}$$

To enhance feature discrimination, we apply a learnable bandpass filter in the frequency domain. This filter selectively amplifies certain frequency components that are empirically found to correlate with disease-relevant patterns. The bandpass range is initialized based on prior frequency spectrum analysis of the dataset and optimized during training.

After filtering, the inverse FFT (IFFT) is applied to return the representation to the spatial domain:

(5) $$i(x,y)=\frac{1}{HW}\sum_{u=0}^{H-1}\sum_{v=0}^{W-1}I(u,v)\cdot e^{2\pi i\left(\frac{ux}{H}+\frac{vy}{W}\right)}$$

where the complex coefficient at the frequency index $\left(u,v\right)$ is in the $I\left[u,v\right]$ frequency domain, and $i\left[m,n\right]$ represents the pixel value at the spatial position $\left(m,n\right)$ in the image. M and N respectively represent the height and width of the image, and u and v are frequency indexes, which vary in the range of [0,M−1] and [0,N−1], respectively. In order to simplify the calculation of FFT processed features, we stack the real part and imaginary part of the frequency-domain results as independent features along the channel dimension. If the number of channels of input I is c, the number of channels of output tensor Y is twice as large as the original, which is expressed as follows:

(6) $$Y[c,u,v]=\left\{\begin{array}{l} I_{r}[u,v]\;\mathrm{if}\;c<C\left(\mathrm{Real}\;\mathrm{part}\right)\\ I_{i}[u,v]\;\mathrm{if}\;C\leq c<2C\left(\mathrm{Imaginary}\;\mathrm{part}\right) \end{array}\right.$$

The tensors obtained are considered to be completely computable [28]. To effectively capture inter-pixel correlations, operations including convolution, batch normalization, and Rectified Linear Unit activation is performed in the frequency domain. The processed tensor, represented as Z, is described mathematically as follows:

(7) Z[c,u,v]=ReLU(BN(Conv(Y[c,u,v])))

In the final step, the tensor  Z is divided along its auxiliary dimension to extract and separate its real and imaginary components and recombined into the complex form. Then, the two-dimensional inverse FFT is applied to the tensor Z to generate an output tensor with a real value, which is calculated as follows:

(8) $$I_{\mathrm{out}}[m,n]=\frac{1}{MN}\sum_{u=0}^{M-1}\sum_{v=0}^{N-1}e^{j2\pi \left(\frac{un}{M}+\frac{vm}{N}\right)}$$

The two-dimensional inverse Fast Fourier Transform reconstructs the spatial-domain representation of the image by aggregating the contributions from each pixel’s real and imaginary parts $\;\left(m,n\right)$. The obtained result, denoted as $I_{\mathrm{out}}[m,n]$, is the reconstructed image in the spatial domain following the inverse transformation. Building upon this foundation, spatial and channel attention mechanisms are incorporated to strengthen the representation capability of features from both local and global perspectives.

In order to more effectively model image features at different scales, we designed two 3×3 convolution layers. $I_{l}$ generates $I_{l\to l}$ and $I_{l\to g}$ through two independent 3×3 convolution layers for processing local and semi-global features. $I_{g}$ is processed independently in 3×3 convolution layer and spectrum block to generate $I_{g\to l}$ and $I_{g\to g}$ for processing semi-global and global features. The spectrum block affects the entire feature map by updating the signal value [29] in the spectrum domain. Semi-global information is integrated into the two feature extraction blocks by combining $I_{l\to g}$ with $I_{g\to g}$, and $I_{g\to l}$ with $I_{l\to l}$, as illustrated below:

(9) $$Y_{1}=f_{g\to l}+f_{l\to l}$$

(10) $$Y_{2}=f_{l\to g}+f_{g\to g}$$

Finally, we combine $Y_{1}$ and $Y_{2}$ through batch standardization and ReLU activation function. Next, the spatial attention mechanism emphasizes global features across each feature map within $Y_{2}$, resulting in $Y_{g}$, while the channel attention mechanism highlights inter-channel relationships within $Y_{1}$, generating $Y_{l}$. Ultimately, the final output $\left\{Y_{g},Y_{l}\right\}$ comprises the combination of $Y_{g}$ and $Y_{l}$.

#### 2.2.2. Cross Vision–Language Alignment Module (CVLA)

**Figure 5:**
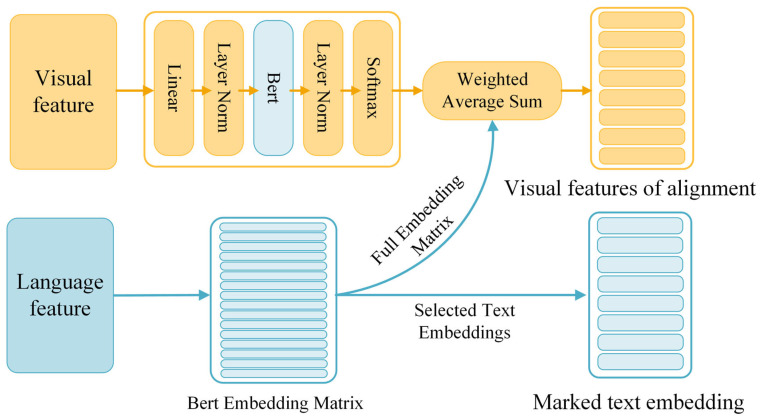
Cross Vision–Language Alignment module.

To enhance the accuracy of tomato disease identification by effectively fusing visual and textual features, we introduce the Cross Vision–Language Alignment (CVLA) module. This module allows the model to capture complementary information from both modalities, improving its recognition capability. Figure 5 illustrates the architecture of the CVLA module, which plays a pivotal role in aligning the image and textual features.

**Figure 6:**
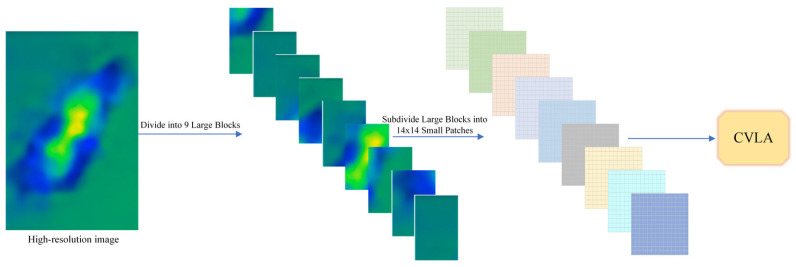
Preprocessing steps of high-resolution image.

As shown in Figure 6, before feeding the images into the CVLA module, we apply a series of preprocessing steps to standardize and optimize the data for model training. To process high-resolution images with varying aspect ratios, we first divide each input image into multiple large blocks based on predefined aspect ratios, selecting the division ratio by coverage as described in [30]. This ensures that the significant regions of the leaf are effectively captured.

To optimize GPU memory usage and maintain computational efficiency, we limit the number of large blocks to nine. Each large block is then subdivided into a grid of small patches, which are projected into vectors and processed by the visual encoder to extract contextual visual features as in [31]. This fixed block and patch scheme guarantees a constant input size for the visual encoder, ensuring stable convergence and consistent feature scales across all disease classes.

By capping the large block count at nine, we prevent generating an excessive number of visual tokens while still capturing sufficient detail from the high-resolution leaf. The 14×14 subdivision provides receptive fields on the order of pixels at the original resolution, allowing the model to capture fine lesion characteristics such as shape, texture, and margin. The choice of a 14×14 subdivision is based on balancing feature granularity and computational cost. This resolution provides pixel-level receptive fields for high-resolution input, enabling the model to detect fine-grained lesion details such as shape irregularities, color variation, and marginal blurring. Compared to other subdivisions like 8 × 8 or 16 × 16, our empirical study found that 14×14 achieves optimal accuracy with minimal memory overhead.

Moreover, averaging the aspect ratios of all large blocks during preprocessing normalizes the input grid structure across images of different scales and capture conditions. This mitigates the influence of irregular shapes or camera distortion, ensuring more consistent visual representations.

Each block $t\in \left\{1,\ldots ,T\right\}$ is divided into $N_{t}$ small blocks, where $P_{t,i}$ is the *i*-th small block of block t.

(11) $$P_{t}=\left\{p_{t,1},\ldots ,p_{t,N_{t}}\right\}$$

Then, the visual encoder maps these image blocks to a set of visual feature vectors:

(12) $$V_{t}=\mathrm{VisionEncoder}\left(P_{t}\right)$$

(13) $$V_{t}=\left\{v_{t,1},\ldots ,v_{t,N_{t}}\right\},v_{t,i}\in R^{d}$$

Finally, we concatenate the feature sets of all blocks into a single output, which are then aligned with the BERT model’s label embedding space through a series of processing steps.

(14) $$F=\mathrm{concat}\left(F_{1},F_{2},\ldots ,F_{T}\right)$$

At the junction of image text output features, we will add the CVLA to achieve alignment between visual features and BERT language models. Specifically, first, through a linear layer $L_{1}\in R^{D\times d}\;$, the visual feature $V\in R^{T\cdot N_{t}\times d}$ is projected into the BERT’s tag embedding space, so that each visual feature corresponds to a vector of $R^{D}$, similar to a token in BERT. Next, the second linear layer $L_{2}\in R^{V\times D}\;$, initialized using the BERT language model header, and the module processes these projected features and subsequently applies an operation to produce the probability distribution $P_{\mathrm{vocab}}\;$, representing the probability of each label of these visual features in the BERT vocabulary.

(15) $$P_{\mathrm{vocab}}=\mathrm{softmax}\left(\mathrm{LayerNorm}\left(L_{2}\cdot \mathrm{LayerNorm}\left(L_{1}V\right)\right)\right)$$

Then, we use BERT’s text embedding $T_{\mathrm{text}}\in R^{V\times D}\;$, which is the embedding vector of each tag in the BERT vocabulary, to calculate the weighted average sum of these embedding vectors. This weighted sum will help the model combine visual and textual features to further achieve cross-modal information fusion. Equation (16) represents calculating the aligned visual feature $V_{\mathrm{align}}^{\prime }$ by multiplying the transpose of the probability distribution $P_{\mathrm{vocab}}$ with the text embedding $T_{\mathrm{text}}$ of BERT.

(16) $$V_{\mathrm{align}}^{\prime }=P_{\mathrm{vocab}}^{⊤}T_{\mathrm{text}}$$

where $T_{\mathrm{text}}(x)\;$ is obtained by tokenizing the input text $x=(x_{1},\ldots ,x_{M})\;$ and selecting the corresponding embedding vectors from $T_{\mathrm{text}}$. Specifically,

(17) $$T_{\mathrm{text}}(x)=\left(T_{\mathrm{text}}(x_{1}),\ldots ,T_{\mathrm{text}}(x_{M})\right).$$

Finally, we concatenate the aligned visual features $V_{\mathrm{align}}^{\prime }$ with the tokenized text embeddings as the input for the classifier. Through CVLA, visual information is transformed into a representation compatible with the BERT language model, enabling effective comparison and integration of visual and linguistic information in the same space. This significantly boosts the model’s capability to interpret cross-modal information, thereby improving its accuracy in recognition tasks.

(18) $$C_{\mathrm{input}}=\mathrm{concat}\left(V_{\mathrm{align}}^{\prime },T_{\mathrm{text}}(x)\right)$$

#### 2.2.3. Multi-Strategy Improved Coati Optimization Algorithm (MSCOA)

In order to obtain better model training effect, we need to carefully select and adjust the learning rate. Therefore, we use the Coati Optimization Algorithm (COA) to find the optimal learning rate. The COA draws inspiration from the coati’s natural predation tactics against iguanas [32]. However, it is easy for only this step to fall into local optimization or deviate from the target. Therefore, we combine the Good Point Set [33] and the Golden Sine Algorithm [34] with the Coati Optimization Algorithm, and propose a Multi-strategy Improved Coati Optimization Algorithm (MSCOA).

Within MSCOA, each coati’s location corresponds to a potential solution. At the initial stage of MSCOA execution, the initialization of positions within the search space is performed randomly based on Equation (19).

(19) $$X_{i}\;∶\;x_{i,j}=lb_{j}+r\cdot \left(ub_{j}-lb_{j}\right),i=1,2,\ldots ,N,j=1,2,\ldots ,m$$

Here, $X_{i}$ indicates the position of the *i*-th coati within the search space corresponding to the $x_{i,j}$ decision variable. Additionally, N represents the total number of coatis, and m denotes the count of decision variables. r symbolizes a random real number uniformly distributed within the interval [0, 1], while $lb_{j}$ and $ub_{j}$ refer to the lower and upper boundaries of the *j*-th decision variable, respectively.

However, COA is prone to uneven population distribution through random initialization of the starting population, which will reduce population diversity and the population quality, thereby influencing the algorithm’s convergence rate. Therefore, we introduce a Good Point Set to improve the method of initializing the population. Let $G_{D}$ be the unit cube in d-dimensional Euclidean space; if $r\in G_{D}$ aube in d-dimensional Euclidean spacets $\phi \left(n\right)=C\left(r,\varepsilon \right)n^{-1+\varepsilon }$, the form is as follows:

(20) $$p_{n}\left(k\right)=\left\{\left(\left\{r_{1}^{\left(n\right)}\times k\right\},\left\{r_{2}^{\left(n\right)}\times k\right\},\ldots ,\left\{r_{i}^{\left(n\right)}\times k\right\}\right),1\leq k\leq n\right\}$$

Then, $p_{n}\left(k\right)$ is called a set of good points, r is a good point, where $C\left(r,\varepsilon \right)$ is a constant only related to r,ε and P is the minimum prime number satisfying $\left(P-D/2\right)\geq D$. It has been proven theoretically that the weighted sum of n good points is smaller than the error obtained by using any other n points and is especially suitable for approximate calculation in high-dimensional space.

Population updates in the search space are guided by a simulation of coati predation on iguanas. Because the Golden Sine Algorithm can traverse all points on the sine function, that is, all points on the unit circle, based on the connection between the unit circle and the sine function, it has strong global search ability, and GSA introduces the golden partition coefficient in its position update process, so that the algorithm will fully search the area that can produce excellent solutions in each iteration process, thus speeding up the convergence speed of the algorithm and jumping out of the local optimum [35]. Therefore, the Golden Sine Algorithm is integrated into the exploration phase of MSCOA in this paper. The GSA simulates the movement of a coati chasing an iguana by mapping positions along a unit circle using the sine function, enabling the algorithm to traverse a wide range of the search space. The key idea is to use the golden ratio $\phi =\frac{1+\sqrt{5}}{2}\approx 1.618$ as a partition coefficient in the update formula. This coefficient guides the position update toward globally optimal regions by biasing exploration toward regions with high solution quality.

Specifically, the mathematical model of tree-climbing coati behavior is given in Equation (21). In this formula, R1 is a random angle for sinusoidal modulation, $r_{1}\in [0,1]$ controls the exploration range, $r_{2}\in [0,1]$ generates diverse angular offsets using $2\pi r_{2}$, and I is a control coefficient balancing attraction to the best solution versus the individual’s own trajectory.

(21) $$x_{i}^{t+1}\left(j\right)=x_{i}^{t}\left(j\right)\cdot \left|\sin R_{1}\right|+r_{1}\sin\left(2\pi r_{2}\right)\left(x_{best}^{t}\left(j\right)-I\cdot X_{i}^{t}\left(j\right)\right),i=1,2,\ldots ,\frac{N}{2}$$

The sine terms ensure that the algorithm explores the unit circle, fully utilizing the search space in a non-linear and oscillatory fashion. The golden ratio is implicitly introduced through the sinusoidal periodicity and random modulation, helping the model oscillate around promising regions and avoid premature convergence.

The landing position of iguanas is random, and the coati on the ground will move randomly accordingly. The mathematical model is as follows:

(22) $$Iguana_{ground}^{t}\left(j\right)=lb+r\cdot \left(ub-lb\right)$$

(23) $$x_{i}^{t+1}\left(j\right)=\left\{\begin{array}{c} x_{i}^{t}\left(j\right)\cdot \left|\sin R_{1}\right|+r_{1}\sin\left(2\pi r_{1}\right)\left(Iguana_{ground}^{t}\left(j\right)-I\cdot x_{i}^{t}\left(j\right)\right),if\;fitness\left(Iguana_{ground}^{t}\right)<fitness\left(x_{i}^{t}\right)\\ x_{i}^{t}\left(j\right)\cdot \left|\sin R_{1}\right|+r_{1}\sin\left(2\pi r_{2}\right)\left(Iguana_{ground}^{t}\left(j\right)-I\cdot x_{i}^{t}\left(j\right)\right),else \end{array},\right.i=\frac{N}{2}+1,\frac{N}{2}+2,\ldots ,N$$

If the new position calculated for each coati increases the value of the objective function, the update process is acceptable; otherwise, the coati will remain in its previous position. This update condition applied to i = 1,2,…,N is simulated with (24).

(24) $$X_{i}=\left\{\begin{array}{l} X_{i}^{P1},F_{i}^{P1}<F_{i}\\ X_{i},else \end{array}\right.$$

Once the positions of all coatis within the search space are updated, an iteration cycle of MSCOA concludes. Following the completion of the MSCOA execution, the optimal solution found throughout the iterative procedure is provided as the final result. The optimal learning rate found with the help of the MSCOA algorithm can better train our model. Additionally, incorporating the Good Point Set improves global search uniformity, while integrating the Golden Sine Algorithm strengthens global exploration capabilities and expedites convergence. These enhancements prevent the algorithm from becoming trapped in local optima, resulting in a more efficient optimization process in high-dimensional spaces and significantly elevating the overall quality and efficiency of solutions.

## 3. Experimental Results and Analysis

### 3.1. Environment and Settings

To minimize variations resulting from differing experimental conditions on FCMNet’s performance, all experiments conducted in this research utilized a standardized computing environment, both in terms of hardware and software configurations. The core hardware setup was provided by AutoDL, a computational platform that supported our experiments with necessary hardware resources, and the software environment included standardized development tools and operating system versions to ensure uniformity across experiments. Specific details of hardware configurations and software settings used in these experiments are summarized in Table 1.

Prior to feeding data into the model, images were cropped and rotated as preprocessing steps. Specifically, the images were cropped to remove unnecessary background and focus on the region of interest, followed by rotation to standardize the orientation of all images, ensuring consistency in the input data before training. This rotation step helped to eliminate any potential bias introduced by varying image orientations in the dataset. Meanwhile, textual data was encoded using a pretrained encoder, standardized in length, and augmented with masking strategies. Subsequently, the dataset was divided into training, validation, and testing subsets according to an 8:1:1 proportion. Specifically, training and validation subsets were used to refine model parameters and weights, while the test subset served to evaluate model performance and generalization capabilities.

Prior to initiating the training process, we utilized the MSCOA algorithm to conduct a preliminary search for the best-performing learning rate. Specifically, the pretraining objective served as the optimization target for MSCOA, with the number of coatis set to 10 and iteration count fixed at 5. The search boundaries for the learning rate ranged from 1 × 10^−6^ to 1 × 10^−2^. Through iterative MSCOA optimization, we identified the optimal learning rate. Subsequently, in the formal training stage, a batch size of 64 was chosen, and although the initial learning rate was initially set at 0.001, it was subsequently updated with the optimal value obtained via MSCOA. The training proceeded for 200 iterations using the Adam optimizer, with a weight decay factor set at 0.01.

### 3.2. Evaluation Indicators

In order to comprehensively evaluate the performance of FCMNet in tomato leaf disease classification, the following four criteria were used: Accuracy, Precision, Recall, and F1 score. These metrics enable an in-depth evaluation of the model’s effectiveness in classifying tomato leaf diseases, especially its classification accuracy and error distribution in different categories. First, four sample definitions are introduced: TN (true negatives) indicates the number of correctly classified negative samples; TP (true positives) refers to the number of correctly identified positive samples; FN (false negatives) represents positive samples incorrectly identified as negative; and FP (false positives) denotes negative samples mistakenly classified as positive.

(25) $$\mathrm{Accuracy}=\frac{\mathrm{TP}+\mathrm{TN}}{\mathrm{TP}+\mathrm{TN}+\mathrm{FP}+\mathrm{FN}}\times 100\%$$

(26) $$\mathrm{Precision}=\frac{\mathrm{TP}}{\mathrm{TP}+\mathrm{FP}}\times 100\%$$

(27) $$\mathrm{Recall}=\frac{\mathrm{TP}}{\mathrm{TP}+\mathrm{FN}}\times 100\%$$

(28) $$F1\text{-}\mathrm{Score}=2\times \frac{\mathrm{Precision}\times \mathrm{Recall}}{\mathrm{Precision}+\mathrm{Recall}}$$

The confusion matrix serves as a tool to visualize the model’s classification performance per category, and helps to analyze the type and distribution of misclassification by displaying the TP, TN, FP, and FN situation of each category in the form of a matrix as follows:

(29) $$\left(\begin{array}{cc} TP & FP\\ FN & TN \end{array}\right)$$

### 3.3. Module Effectiveness Experiment

To validate the effectiveness of the FGAM, CVLA, and MSCOA modules within our proposed model, we performed comprehensive experiments. This section analyzes how each module contributes individually to model performance, presenting detailed experimental results across different configurations.

#### 3.3.1. Effectiveness of FGAM

In this study, we propose a novel attention mechanism, FGAM, which introduces the Fourier transform into the spatial-channel attention structure to enhance the stability and noise immunity of the features and improve the perception of the diseased spot region under complex backgrounds. The module divides local and global features by separating factors, combines convolutional and frequency-domain information to realize multi-scale information fusion, and effectively improves the robustness and detail recognition ability of the model. In order to verify the effectiveness of FGAM, we used ResNet50 as the baseline model [36] and compared it with three existing attention mechanisms: efficient channel attention (ECA) [37], mild triple-disease focused attention (MTDFA) [38] and convolutional attention module (CBAM) [39], and the experimental results are shown in Table 2. With the addition of ECA and MTDFA attentional mechanisms, the accuracy and F1 scores of ResNet50 decreased, while other evaluation metrics were slightly improved. In contrast, all evaluation metrics are improved after adding CBAM and FGAM attention mechanisms, with the most significant improvement in FGAM, which improves 0.78%, 0.91%, 1.06%, and 1.14% in accuracy, precision, recall, and F1 score, respectively. Specifically, ECA improves the channel feature expression ability while keeping the model lightweight by introducing the local interaction strategy, but it neglects the spatial information modeling, which makes it difficult to capture the spatial distribution characteristics of the plant spots in the image and affects the recognition effect. MTDFA performs better in the local feature extraction, which is especially suitable for minor diseases, but it mainly relies on the convolution operation to construct the spatial attention, which is not suitable for the global feature modeling in complex backgrounds, which is still insufficient and prone to interference. CBAM combines spatial and channel attention, which helps to highlight key regions in the image, but has limitations in dealing with fine-grained differences or global semantic modeling, and performs erratically especially when the boundary of the lesion is blurred or the morphology is not clear. In contrast, FGAM effectively fuses spatial, channel and spectral information by introducing Fourier frequency-domain features, which enhances the global robustness and noise immunity of the model while enhancing the detail perception, and demonstrates stronger comprehensive recognition performance.

#### 3.3.2. Effectiveness of the CVLA

Existing multimodal feature fusion methods typically rely on straightforward concatenation of features from different modalities. In contrast, our approach integrates the CVLA module specifically designed to enhance the fusion of image and textual features for tomato disease recognition, thereby promoting deeper interactions and improved cross-modal understanding. In order to verify the effectiveness of CVLA, we compared CVLA with three other advanced cross-modal alignment modules such as FiLM [40], GMU [41] and CMAT [14] to fully evaluate the effectiveness of the CVLA module, and we chose ResNet50 and TinyBert [42] for the extraction of visual features and textual features, respectively. According to the results presented in Table 3, CVLA outperforms other cross-modal alignment methods in all evaluation metrics and shows significant improvement. Compared with the baseline model ResNet50 + BERT, CVLA improves 1.16%, 1.24%, 1.31%, and 1.41% in accuracy, precision, recall, and F1 score, respectively. Although FiLM, GMU and CMAT improve in some indicators, their comprehensive performance is still inferior to that of CVLA; specifically, the gap in recall rate and F1 score is large. CVLA can achieve such an effect mainly due to the following points: Firstly, through the block partitioning strategy and visual feature encoding, CVLA is able to capture the local lesion features in the image in a more detailed way, while maintaining the overall image structure information, which provides more accurate visual representation for the subsequent cross-modal alignment. Secondly, CVLA adopts a mapping method compatible with BERT embedding, which makes it easier to align visual features with text in the semantic space, and makes up for the lack of interaction at the semantic level in the traditional splicing fusion method. In addition, the introduced probabilistic weighting mechanism further strengthens the higher-order semantic correlation between image and text, enabling the model to dynamically adjust the contribution ratio of different modal information, thus improving the overall feature fusion quality.

#### 3.3.3. Effectiveness of the MSCOA

To evaluate the effectiveness of the proposed Multi-strategy Improved Coati Optimization Algorithm (MSCOA), we performed comparative experiments involving several other optimization methods. Specifically, ResNet50 was utilized for image feature extraction and TinyBERT served as the text feature extractor. The comparative results, presented in Table 4, demonstrate that MSCOA outperforms alternative algorithms across all evaluation metrics, achieving accuracy, precision, recall, and F1 scores of 95.91%, 95.73%, 95.50%, and 95.56%, respectively. Compared to the baseline model, MSCOA has improved by 0.48%, 0.59%, 0.72%, and 0.67%, respectively, showing significant performance improvement. Although the standard COA already outperforms other optimization algorithms, such as GWO [43], WOA [44], and PSO [45], MSCOA further improves on its foundation, especially in recall and F1 score, which is more obvious, suggesting that it has stronger adaptive and generalization capabilities when dealing with boundary samples and fuzzy features. The enhanced performance of MSCOA stems from two key modifications to the original COA. Firstly, incorporating the Good Point Set method enhances population initialization uniformity, leading to more comprehensive coverage of the search space and improved exploration efficiency during the algorithm’s initial phase. Secondly, integrating the Golden Sine Algorithm boosts global exploration capabilities, leveraging the golden ratio strategy to effectively prevent premature convergence to local optima and further expedite convergence. Compared with traditional optimization algorithms, COA may be biased in the early stage of the search, GWO and WOA are prone to fall into local optimum in the late iteration, and PSO has stable convergence but slower overall optimization speed. In contrast, MSCOA achieves a better balance between global exploration and local exploitation, which is particularly suitable for high-dimensional complex optimization tasks and provides a more efficient and stable solution for deep network parameter search.

### 3.4. Ablation Experiments

To systematically assess how each enhancement module affects the performance of the FCMNet architecture, we conducted a series of ablation experiments. The detailed results of these experiments are summarized in Table 5. Three modules are introduced in the experiments: the FGAM, the CVLA, and the MSCOA. We conducted eight sets of detailed ablation experiments by controlling variables based on Resnet50 + TinyBert. The experimental results show that the CVLA module performs well in multimodal feature alignment and significantly improves the model in various performance metrics such as accuracy, precision, recall, and F1 score. The module effectively enhances the comprehensive interaction between linguistic and visual modalities through the image block division and feature mapping mechanism, thus improving the accuracy of cross-modal semantic understanding. The FGAM module, as an improved attention mechanism, introduces the Fourier transform into the spatial-channel attention structure, which strengthens the stability of the feature expression and the noise resistance, and enables the model to more accurately identify the lesion area under complex backgrounds. The accuracy of attention allocation and the robustness of feature extraction are effectively improved. In addition, the MSCOA optimization algorithm improves the efficiency of the learning rate search and the ability to obtain the global optimal solution by introducing the Good Point Set and the Golden Sine strategy, which enhances the model’s capability in handling high-dimensional optimization tasks, while simultaneously speeding up the network’s convergence. In summary, CVLA, FGAM and MSCOA each enhance the model from different perspectives and play key roles in modal fusion, attention mechanism and training optimization, respectively, and the three synergistically work together to promote the performance of FCMNet.

### 3.5. Experiment Comparing FCMNet with Other Models

**Figure 7:**
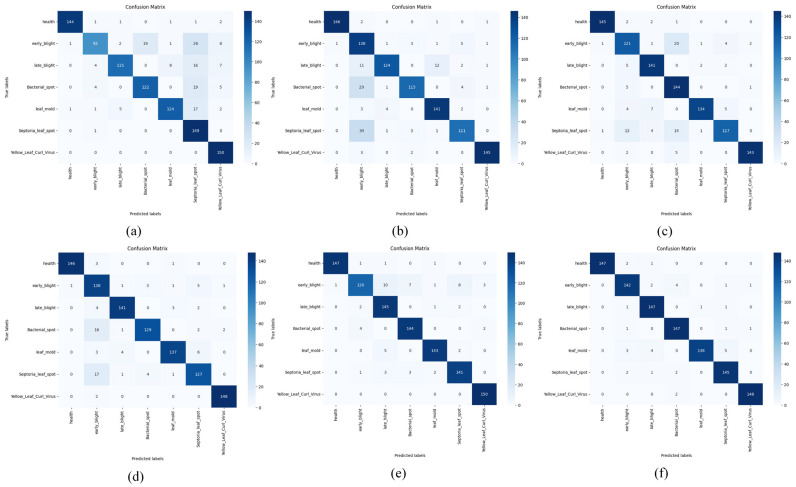
Confusion matrix of recognition results of (**a**) MobilenetV3, (**b**) MobileViT, (**c**) DINOV2, (**d**) ResNet50, (**e**) HCA-MFFNet and (**f**) FCMNet.

To demonstrate the superiority of FCMNet performance, it was compared with MobilenetV3 [46], MobileViT [47], DINOV2 [48], ResNet50, and HCA-MFFNet [18] to evaluate the performance of FCMNet for tomato leaf disease classification. All experiments were conducted on the same test set, and the results are shown in Table 6 and Figure 7.

According to the experimental outcomes, FCMNet attains the highest performance compared to other models, reaching an accuracy level of 98.04%, and improves significantly compared to MobilenetV3′s 78.67%, MobileViT’s 88.27% and DINOV2′s 89.05%, and outperforms ResNet50′s 95.43% and HCA-MFFNet’s 96.12%. Meanwhile, the precision and recall of FCMNet were 97.99% and 97.81%, respectively, which were significantly better than the other models, indicating that it not only effectively reduces the misclassification, but also maximizes the capture of disease samples. Combining all the indexes, the F1 value of FCMNet reached 97.95%, demonstrating an effective balance between precision and recall, thereby offering stable and dependable technical support for detecting tomato leaf diseases. To further explore the underlying reasons for the superior performance of FCMNet compared with other models, we propose the following hypotheses:

1. FCMNet uses ResNet50 as the benchmark backbone network, and this framework has demonstrated excellent performance and stability in several image classification tasks. ResNet50 effectively mitigates the gradient vanishing and degradation problems that occur during the training of deep neural networks through the introduction of Residual Connection, which enables the network to achieve highly efficient training while maintaining a deeper number of layers. Residual connections effectively alleviate the issues of gradient disappearance and network degradation commonly encountered during deep neural network training, which enables the network to realize efficient training while maintaining a deeper number of layers [49].
2. We constructed a dataset containing 6994 tomato leaf images, including both diseased and healthy leaf samples, and equipped the images with detailed text descriptions, which provides rich semantic context for the model, contributes to the deep fusion between visual and linguistic modalities, and strengthens the model’s discriminative and generalization abilities.
3. The Fourier-guided Attention Mechanism (FGAM) introduced by FCMNet embeds Fourier frequency-domain features in the spatial-channel attention structure, which not only improves the model’s attention to the diseased spot region, but also enhances the stability and noise resistance of feature expression by fusing the local and global features. The FGAM effectively improves the robustness of the model and the ability of detail extraction in complex contexts.
4. In terms of multimodal fusion, our proposed CVLA module maps visual features to a semantic space compatible with text embedding through image block partitioning and feature encoding, and strengthens cross-modal alignment through a probabilistic weighting mechanism, which effectively improves the depth of the information interaction between image and text, thus enhancing the model’s semantic comprehension ability.
5. During the training stage, we propose a Multi-strategy Improved Coati Optimization Algorithm (MSCOA), which incorporates the Golden Sine Algorithm along with the Good Point Set strategy to optimize the search process for the learning rate. This approach effectively enhances convergence speed, boosts global search efficiency, prevents the model from becoming trapped in local optima, and ultimately leads to superior model performance in reduced training time.

### 3.6. Generalization Experiments

To evaluate the generalization capability of the proposed FCMNet model, we conducted cross-dataset validation using the publicly available Maize Leaf Disease Spectrum (MLDS) dataset [14]. Unlike our primary tomato leaf dataset, MLDS features a distinct crop species (maize) and different disease manifestations, making it suitable for assessing cross-domain robustness. The dataset comprises 4633 images spanning three maize diseases—Fusarium wilt, rust, and gray leaf spot—as well as one healthy class. Each image is paired with a corresponding textual description, maintaining a multimodal structure consistent with our framework’s design.

MLDS poses unique challenges due to the subtle visual differences among disease types and the potential confusion between pest and disease symptoms. These characteristics make it a strong benchmark for testing the model’s capacity to generalize to unseen disease categories and a new crop context.

We compared our model’s performance with a state-of-the-art method specifically designed for maize disease classification, WCG-VMamba, and summarized the results in Table 7. The FCMNet model achieves competitive performance on MLDS, with an F1 score of 96.07%, slightly outperforming WCG-VMamba in terms of F1 despite marginally lower accuracy. This indicates that FCMNet can effectively adapt to new domains and maintain strong discriminative power even in unfamiliar visual environments.

These findings provide empirical evidence that the proposed model is not only effective on in-domain tomato leaf disease tasks but also exhibits strong cross-domain generalization, highlighting its potential for broader application across diverse agricultural scenarios.

### 3.7. Comparison of Visualization Results

Although the multimodal FCMNet model achieved excellent classification performance in the task of identifying tomato leaf diseases, the feature selection mechanism and decision basis within the model still lack intuitive explanation. To improve the model’s interpretability and further understand its mechanism of focusing on key regions in the image, we utilized the Gradient-weighted Class Activation Mapping (Grad-CAM) technique [50] to visualize and analyze the model’s regions of interest (ROI), thereby enhancing its interpretability. Grad-CAM serves as a method for visual interpretation based on the generation of gradient information by calculating the gradient of the classification result to a specific convolutional layer feature map, reflecting the degree of contribution of each region in the image to the final prediction. The generated thermal map can highlight the image areas that the model pays most attention to when making classification decisions, that is, pixels that significantly influence the task of classification. The closer the color of the thermal map to red, the more critical the region is in model judgment. The closer the color to blue, the smaller the impact.

We compared FCMNet’s heatmaps with those generated by the HCA-MFFNet model, and the results are shown in Table 8. For each disease category, representative Grad-CAM visualizations are provided to illustrate differences in attention distribution. In the case of Early Blight, FCMNet consistently concentrated on lesion boundaries and necrotic textures, while HCA-MFFNet often misdirected attention toward healthy leaf areas. For Late Blight, FCMNet effectively localized large irregular lesion areas, whereas HCA-MFFNet’s activation was primarily concentrated along the leaf edges, failing to accurately focus on the core infected regions. This limitation reduced its interpretability and classification accuracy for this category. In Bacterial Spot, FCMNet captured the scattered dark spots more precisely, showing tighter focus on symptomatic clusters. For Leaf Mold, FCMNet emphasized the subtle yellowing and mold patches along the veins, which were less discernible in HCA-MFFNet’s maps. In Septoria Leaf Spot, FCMNet highlighted the small, circular lesions with clear margins, whereas HCA-MFFNet exhibited inconsistent focus across samples. Lastly, for Yellow Leaf Curl Virus, FCMNet showed enhanced sensitivity to leaf curling patterns and chlorosis distribution, a distinction that was less evident in the baseline model.

These detailed visualizations demonstrate FCMNet’s superior ability to locate fine-grained, disease-specific patterns, attributed to the incorporation of the Fourier-guided Attention Mechanism (FGAM) and Cross Vision–Language Alignment (CVLA), which collectively enhance spatial localization and semantic consistency. Additionally, the Multi-strategy Improved Coati Optimization Algorithm (MSCOA) contributes to more efficient convergence and robust feature learning.

Moreover, the Grad-CAM results also indicate potential avenues for improvement. For example, in cases where lesion boundaries blend into healthy tissue, the attention may slightly diffuse, suggesting the need for more diverse data augmentation strategies or inclusion of higher-resolution lesion annotations during preprocessing. These findings offer valuable feedback for refining both the model architecture and data pipeline, contributing to more reliable deployment of FCMNet in practical agricultural settings.

### 3.8. Model Efficiency and Deployment Feasibility

In this section, we evaluate the computational efficiency and feasibility of deploying FCMNet on resource-constrained devices, such as mobile plant protection equipment, by considering key performance metrics like computational complexity, inference time, and memory requirements. The results presented in Table 6 and Table 9 are used to compare FCMNet with other state-of-the-art models in terms of both performance and computational efficiency.

FCMNet demonstrates superior performance in terms of classification accuracy, precision, recall, and F1 score, as shown in Table 6. It achieves an accuracy of 98.04%, which is significantly higher than HCA-MFFNet (96.12%) and other models such as ResNet50 (95.43%) and DINOV2 (89.05%). The precision (97.99%), recall (97.81%), and F1 score (97.95%) of FCMNet further reflect its ability to classify tomato leaf diseases with high reliability, making it highly suitable for real-world applications in plant disease detection.

In terms of computational complexity and inference time, FCMNet strikes a balance between performance and efficiency. Table 9 shows that FCMNet has 16.8 million parameters, which is smaller than DINOV2 (86.1 M) and HCA-MFFNet (31.2 M). This makes FCMNet a more compact model that is easier to deploy on devices with limited computational resources. Additionally, FCMNet has a low average latency of 18.86 ms, compared to ResNet50 (223.05 ms) and HCA-MFFNet (191.23 ms). This low latency allows FCMNet to deliver real-time predictions, which is critical for time-sensitive applications such as plant disease detection.

FCMNet also performs well in terms of total inference time, taking 3:30:39 to process a given dataset, which is faster than DINOV2 (5:54:24) and HCA-MFFNet (4:11:32). Despite the lower inference time, FCMNet maintains high accuracy, making it suitable for applications that require rapid processing of large numbers of images, such as mobile plant protection systems in the field.

Regarding memory usage, FCMNet requires 573.4 MB of peak memory, which is relatively efficient compared to models like HCA-MFFNet (650.4 MB) and DINOV2 (502.8 MB), while being comparable to MobilenetV3 (566.7 MB). This indicates that FCMNet can be deployed on devices with moderate memory capacities, making it feasible for use in mobile plant protection equipment with limited resources.

Finally, the model size of FCMNet is 634.3 MB, which is larger than MobilenetV3 (115.2 MB), but smaller than HCA-MFFNet (845.8 MB) and DINOV2 (713.6 MB). Although FCMNet’s model size is relatively large, it remains manageable compared to other state-of-the-art models, and optimization techniques such as quantization and model compression can be applied to further reduce its size for deployment on mobile devices.

## 4. Discussion

To thoroughly assess the performance of FCMNet in recognizing tomato diseases, we conducted comparative experiments between FCMNet and other plant pest and disease detection models: MobilenetV3, MobileViT, DINOV2, ResNet50, and HCA-MFFNet; the results are shown in Table 6. Under the same experimental conditions, FCMNet achieved better experimental results on the self-constructed tomato leaf disease dataset by comparing with MobilenetV3, MOBILEVIT, DINOV2, ResNet50 and HCA-MFFNet. In comparison to the baseline ResNet50 architecture, FCMNet improved accuracy and precision by 2.61% and 2.85% and recall and F1 score by 3.03% and 3.06%, respectively, providing methodological support for rapid identification and detection of tomato leaf diseases.

Figure 7 demonstrates the confusion matrix results of MobileNetV3, MobileViT, DINOV2, ResNet50, HCA-MFFNet, and FCMNet in the tomato leaf disease classification task. The analysis shows that FCMNet exhibits higher accuracy and robustness in the recognition of all types of diseases. In contrast, MobileNetV3 performs poorly in recognizing multiple disease categories, specifically showing the lowest accuracy on the Early Blight category and obvious misclassification on Late Blight, Bacterial Spot, and Leaf Mold, which indicates its limited feature extraction capability. MobileViT has the lowest accuracy on the Bacterial Spot, Septoria Leaf Spot and Late Blight with decreased accuracy, indicating that it has some deficiencies in handling fine-grained disease features. DINOV2 performs better than the previous two as a whole, but ResNet50 and HCA-MFFNet still have relatively higher recognition accuracies in each category, and their performance is more balanced. The accuracy of ResNet50 in the categories of Bacterial Spot and Septoria Leaf Spot is still reduced; while HCA-MFFNet outperforms the preceding four models in overall effectiveness, it still does not perform as well as FCMNet on categories such as Early Blight. Taken together, FCMNet achieves a more focused attention distribution with more accurate recognition results on all categories, especially in the classification of confusing diseases such as Early Blight. This is due to the introduction of the Fourier-guided Attention Mechanism (FGAM) to improve the localization of the lesion area, the Cross Vision–Language Alignment module (CVLA) to strengthen the semantic synergy between image and text, and the Multi-strategy Improved Coati Optimization Algorithm (MSCOA) to enhance the convergence efficiency and global optimization ability. This enables FCMNet to have better comprehensive performance in multiple levels, such as feature extraction, information fusion and classification discrimination. The confusion matrix analysis further verifies the stability and practicality of FCMNet in tomato disease identification.

While FCMNet has demonstrated strong performance in classifying most disease categories, there is still potential for improvement in identifying certain diseases. For example, in the case of Late Blight, the attention weights distribution in the disease–healthy junction region, as shown in Table 8, reveals some diffusion, indicating that the model has not yet fully captured the subtle pathological features of Late Blight. This could be due to the typicality of the samples or the complexity of the spot morphology. To address this, future work will focus on enhancing the model’s ability to generalize on fine pathological features by expanding the disease image dataset to include multiple growth stages and varied ambient light conditions.

Additionally, while our model currently relies on both leaf images and textual descriptions for disease recognition, we are also exploring the possibility of using leaf images alone for effective detection in real-world applications. Reducing reliance on textual descriptions could enhance the model’s adaptability in environments where textual data may not be available. Future work will investigate the feasibility of image-only solutions, while maintaining high performance in disease recognition and optimizing the model for lightweight, real-time deployment in mobile plant protection equipment. This would support the broader adoption of crop disease monitoring technology in field conditions and provide reliable technical support for disease early-warning systems in smart agriculture.

Apart from overall classification accuracy, we also examined FCMNet’s performance in handling real-world edge cases, particularly early-stage diseases and heavily occluded leaves, which are common challenges in agricultural scenarios. Early-stage diseases are often difficult to detect due to subtle visual cues and small lesion areas, while occlusions—caused by overlapping leaves, shadows, or background noise—may partially obscure key pathological features.

Experimental observations show that FCMNet maintains relatively high recognition accuracy even under these challenging conditions. For instance, the model outperforms competing architectures in the Early Blight and Septoria Leaf Spot categories, which are prone to misclassification in early or partially visible stages. This advantage is primarily attributed to the Fourier-guided Attention Mechanism (FGAM), which enhances sensitivity to fine-grained patterns and supports accurate lesion localization even when features are weak or spatially limited. Additionally, the Cross Vision–Language Alignment (CVLA) module leverages textual information to supplement visual ambiguities, allowing the model to infer disease identity from context even under partial occlusion.

These results highlight the practical robustness of FCMNet in real-world scenarios where data quality is often imperfect. The model’s resilience to edge cases strengthens its potential for deployment in field conditions, supporting continuous disease monitoring and early-warning systems in precision agriculture.

## 5. Conclusions

Relying solely on image data typically fails to capture all relevant information required for accurate classification of tomato leaf diseases. In contrast, multimodal feature fusion combines visual and textual information, effectively leveraging complementary features to enhance recognition accuracy and model robustness. Consequently, we introduced the FCMNet framework, a multimodal fusion framework tailored for tomato leaf disease identification. Additionally, we built an image dataset comprising 6994 annotated instances of tomato leaf diseases to support our research, and we also equipped the images in the dataset with textual descriptions from experts to better train our network. For our model FCMNet, three innovative modules are employed to accurately recognize tomato leaf diseases. First, we propose the Fourier-guided Attention Mechanism (FGAM), and this is the first time Fourier frequency-domain features are embedded into a spatial-channel attention structure. This significantly enhances the model’s robustness in capturing diseased regions against complicated backgrounds and improves the stability of feature representations against noise interference. FGAM constructs a multi-scale complementary information representation through the delineation, and integrating global and local features substantially strengthens the robustness and detail-capturing capability of the model. Secondly, to effectively leverage the semantic associations between textual and visual data, the Cross Vision–Language Alignment module (CVLA) is designed. This module maps the visual information into the space compatible with BERT embedding through image block division and visual feature encoding, and realizes the in-depth fusion of graphical and textual information with the help of probabilistic weighting mechanism, thereby enhancing the model’s capability to capture and represent semantic relationships across different modalities. Finally, in order to improve the optimization efficiency and global optimization ability of the model during the training process, the Multi-strategy Improved Coati Optimization Algorithm (MSCOA) is introduced. The algorithm combines the Good Point Set method and the Golden Sine Algorithm, which enhances the global search ability while improving the uniformity of population initialization, avoids falling into local optimums and accelerates the convergence speed, which is especially suitable for parameter optimization in high-dimensional complex spaces.

In our experiments, we split the data in our self-constructed dataset into training, validation, and test sets in 8:1:1 instances, and use these samples to train FCMNet, among others. The ablation experiments demonstrate the effectiveness of FGAM, CVLA, and MSCOA. Compared with ResNet50, the baseline network of FCMNet, FCMNet improved 2.61% and 2.85% in accuracy and precision, and 3.03% and 3.06% in recall and F1 scores, respectively, and outperformed the baseline model overall, as well as compared with other state-of-the-art disease recognition models. Our model also outperformed other state-of-the-art disease recognition models in accuracy, precision, recall, and F1 score, respectively, 98.04%, 97.99%, 97.81%, and 97.95%, which exceeded the performance of existing models.

Looking ahead, two key directions are proposed for future work. First, we plan to expand the current dataset in terms of both scale and diversity by incorporating a wider range of real-world samples collected under varied environmental conditions and from different cultivars. This effort aims to enhance the generalization ability and robustness of the model in complex and unpredictable field scenarios. Second, we intend to optimize FCMNet for deployment on resource-constrained platforms such as mobile plant protection devices. This will include techniques such as parameter pruning, model quantization, and the design of more efficient multimodal fusion architectures.

**Figure 8:**
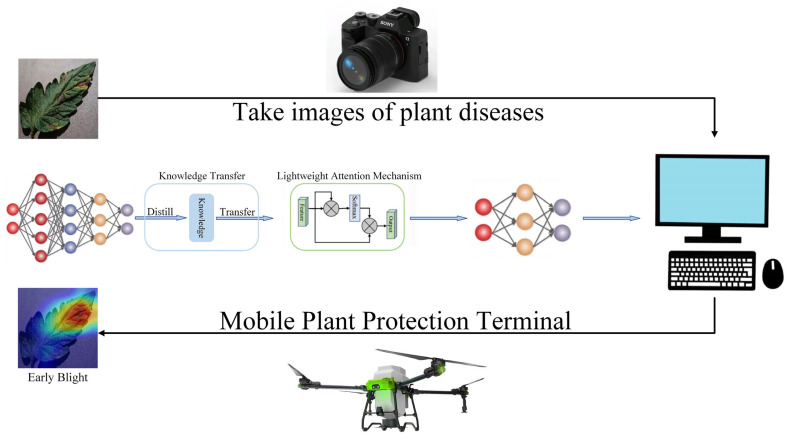
Roadmap for lightweight deployment of FCMNet in resource-constrained environments.

To facilitate this optimization, a potential roadmap is illustrated in Figure 8. This roadmap outlines key strategies including modular distillation of the vision and language processing branches, the adoption of lightweight attention mechanisms, and the implementation of adaptive inference strategies tailored for deployment on mobile plant protection terminals. These efforts aim to significantly reduce model complexity and computational demands while retaining classification performance. However, critical challenges remain: particularly, preserving high recognition accuracy under aggressive compression and ensuring real-time responsiveness on devices with limited memory and processing power. Overcoming these obstacles is essential for enabling the practical, real-world deployment of FCMNet in agricultural environments.

In conclusion, the proposed FCMNet exhibits strong performance in multimodal tomato disease recognition and provides a solid foundation for further developments in intelligent plant disease diagnosis and embedded agricultural AI systems.

## Figures and Tables

**Table 1:** Hardware and software parameters.

Environment	Type	Parameters
**Hardware** **environment**	**CPU**	18 vCPU AMD EPYC 9754 128-Core Processor @ 2.50 GHz
	**GPU**	NVIDIA GeForce KTX 4090D (24 GB)
	**RAM**	80 G
	**Video Memory**	32 G
**Software environment**	**CUDA**	11.6
	**CUDNN**	8.0.5
	**Python**	3.8
	**Pytorch**	1.12.1

**Table 2:** Comparison of the results of different attention mechanisms.

Model	Accuracy	Precision	Recall	F1 Score
ResNet50	95.43%	95.14%	94.78%	94.89%
+ECA	95.31%	95.01%	94.93%	95.11%
+MTDFA	95.47%	95.22%	94.84%	94.73%
+CBAM	95.62%	95.47%	95.36%	95.42%
+FGAM	96.21%	96.05%	95.84%	96.03%

**Table 3:** Comparison of results for different cross-modal alignment modules.

Model	Accuracy	Precision	Recall	F1 Score
ResNet50 + Bert	95.43%	95.14%	94.78%	94.89%
+FiLM	95.49%	95.35%	95.22%	95.14%
+GMU	95.57%	95.29%	95.18%	94.96%
+CMAT	95.82%	96.02%	95.56%	95.92%
+CVLA	96.59%	96.38%	96.37%	96.30%

**Table 4:** Comparison of results of different optimization algorithms.

Model	Accuracy	Precision	Recall	F1 Score
ResNet50 + Bert	95.43%	95.14%	94.78%	94.89%
+GWO	94.21%	94.86%	94.83%	94.65%
+WOA	95.22%	95.04%	94.89%	94.96%
+PSO	95.06%	94.53%	94.68%	94.76%
+COA	95.52%	95.27%	94.91%	95.32%
+MSCOA	95.91%	95.73%	95.50%	95.56%

**Table 5:** Ablation experiment results.

Group	FGAM	CVLA	MSCOA	Accuracy	Precision	Recall	F1 Score
①				95.43%	95.14%	94.78%	94.89%
②	**√**			96.21%	96.05%	95.84%	96.03%
③		**√**		96.59%	96.38%	96.37%	96.30%
④			**√**	95.91%	95.73%	95.50%	95.56%
⑤	**√**	**√**		97.48%	97.31%	97.16%	97.27%
⑥		**√**	**√**	97.15%	97.01%	96.96%	96.82%
⑦	**√**		**√**	96.66%	96.42%	96.34%	96.48%
⑧	**√**	**√**	**√**	98.04%	97.99%	97.81%	97.95%

**Table 6:** Comparison of experimental results.

Model	Accuracy	Precision	Recall	F1 Score
MobilenetV3	78.67%	77.14%	76.61%	78.08%
MobileViT	88.27%	85.57%	85.21%	85.67%
DINOV2	89.05%	88.29%	86.36%	86.89%
ResNet50	95.43%	95.14%	94.71%	94.89%
HCA-MFFNet	96.12%	95.63%	95.71%	96.26%
FCMNet	98.04%	97.99%	97.81%	97.95%

**Table 7:** Results of generalization experiments on the Maize Leaf Disease dataset.

Model	Accuracy (%)	Precision (%)	Recall (%)	F1 (%)
WCG-VMamba	96.97	95.94	96.04	95.99
ours	96.85	95.52	95.81	96.07

**Table 8:** Visualization comparison results.

Model	Leaf Mold	Bacterial Spot	Septoria Leaf Spot	Yellow Leaf Curl Virus	Late Blight	Early Blight
	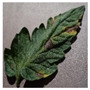	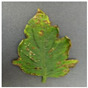	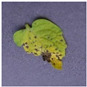	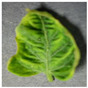	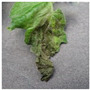	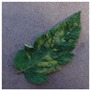
HCA-MFFNet	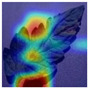	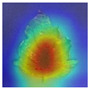	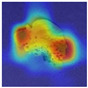	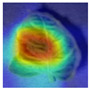	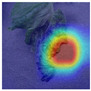	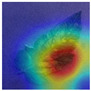
FCMNet	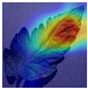	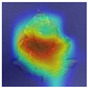	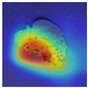	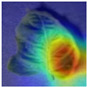	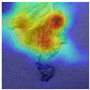	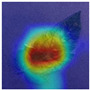

**Table 9:** Comparison of computational efficiency and model size.

Metrics	MobilenetV3	DINOV2	ResNet50	HCA-MFFNet	FCMNet
Params(M)	5.4	86.1	15.6	31.2	16.8
Avg-Latency (ms)	18.64	47.92	223.05	191.23	18.86
Peak Memory (MB)	566.7	502.8	566.1	650.4	573.4
GFLOPs	1.22	4.51	1.33	3.57	1.45
Model Size (MB)	115.2	713.6	598.3	845.8	634.3
Total Inference Time	3:28:10	5:54:24	3:45:01	4:11:32	3:30:39

## Data Availability

The datasets in this study are available upon request from the corresponding author.
